# Wide-ranging transcriptomic analysis of *Poncirus trifoliata*, *Citrus sunki, Citrus sinensis* and contrasting hybrids reveals HLB tolerance mechanisms

**DOI:** 10.1038/s41598-020-77840-2

**Published:** 2020-11-30

**Authors:** Maiara Curtolo, Inaiara de Souza Pacheco, Leonardo Pires Boava, Marco Aurélio Takita, Laís Moreira Granato, Diogo Manzano Galdeano, Alessandra Alves de Souza, Mariângela Cristofani-Yaly, Marcos Antonio Machado

**Affiliations:** 1 Centro de Citricultura Sylvio Moreira, Instituto Agronômico de Campinas, Cordeirópolis, SP Brazil; 2 Universidade Estadual de Campinas, Campinas, SP Brazil

**Keywords:** Biotechnology, Genetics, Molecular biology, Plant sciences

## Abstract

Huanglongbing (HLB), caused mainly by ‘*Candidatus* Liberibacter asiaticus’ (CLas), is the most devastating citrus disease because all commercial species are susceptible. HLB tolerance has been observed in *Poncirus trifoliata* and their hybrids. A wide-ranging transcriptomic analysis using contrasting genotypes regarding HLB severity was performed to identify the genetic mechanism associated with tolerance to HLB. The genotypes included *Citrus sinensis, Citrus sunki, Poncirus trifoliata* and three distinct groups of hybrids obtained from crosses between *C. sunki* and *P. trifoliata.* According to bacterial titer and symptomatology studies, the hybrids were clustered as susceptible, tolerant and resistant to HLB. In *P. trifoliata* and resistant hybrids, genes related to specific pathways were differentially expressed, in contrast to *C. sinensis*, *C. sunki* and susceptible hybrids, where several pathways were reprogrammed in response to CLas. Notably, a genetic tolerance mechanism was associated with the downregulation of gibberellin (GA) synthesis and the induction of cell wall strengthening. These defense mechanisms were triggered by a class of receptor-related genes and the induction of WRKY transcription factors. These results led us to build a hypothetical model to understand the genetic mechanisms involved in HLB tolerance that can be used as target guidance to develop citrus varieties or rootstocks with potential resistance to HLB.

## Introduction

Huanglongbing (HLB) or greening has been considered the most devastating citrus disease. HLB is caused by the gram-negative, phloem-limited, α-proteobacterium *Candidatus* Liberibacter species. The following three Liberibacter species have been associated with HLB: *Candidatus* Liberibacter asiaticus (CLas), *Candidatus* Liberibacter americanus (CLam) and *Candidatus* Liberibacter africanus (CLaf). CLas is the most widespread and is responsible for large economic losses worldwide^1,2^.

HLB symptoms include blotchy chlorosis, mottling of leaves, yellow shoots, vein corking, stunted growth and small, green, and lopsided fruits with aborted seeds^3^. HLB symptom development is considered a consequence of a series of molecular, cellular, and physiological disorders in the plant host. The most expressive modifications caused by CLas in the citrus host are alterations in sucrose and starch metabolism, changes of hormone production, biosynthesis of secondary metabolites, phloem function disorders, and source-sink communication^4,5^.

*Poncirus trifoliata* is closely related and sexually compatible with the citrus genus, and it shows attenuated HLB symptoms and lower CLas titer, indicating that this genus possibly presents genetic defense mechanism against CLas^6,7^. Moreover, some citrus hybrids of *P. trifoliata* have also been reported to present a significant tolerance to HLB^7,8^; however, it remains unclear which mechanisms are involved in this tolerance. In contrast, all commercial *Citrus* species are susceptible to CLas infection, and the identification of tolerant genotypes is essential to the maintenance of citrus production^2^. Studies are still necessary to understand better the differences of genetic responses involved in the susceptibility, tolerance or resistance to such genotypes, aiming to obtain new citrus variety tolerant to HLB by conventional breeding or genetic engineering.

Our study provides a wide-ranging transcriptomic analysis of two CLas-susceptible citrus genotypes (*Citrus sinensis* and *C. sunki*), one CLas-tolerant genotype (*P. trifoliata*), and three pools of hybrids between *P. trifoliata* and *C. sunki*, which are classified as susceptible, tolerant, and resistant to HLB. Therefore, this work was the first to study transcriptional reprogramming and to compare the results of a large volume of transcriptomes, including individuals from a population of hybrids infected by CLas, which consequently inherited the susceptible and tolerance genetic mechanisms from their parents.

The results revealed that only a few genes associated with specific pathways were modulated in resistant genotypes to avoid CLas proliferation and plant disease severity. Using the transcriptomic analysis of the hybrid genotypes, we revalidated the mechanisms of susceptibility and tolerance of their parents. Based on the analysis, we built a hypothetical model to explain the genetic mechanism involved in HLB tolerance conferred by *P. trifoliata* and inherited by its hybrids that could be further used in breeding or biotechnological approaches.

## Results

### CLas quantification

CLas quantification analysis showed that all plants from *C. sinensis*, *C. sunki*, and *P. trifoliata* were infected by CLas after 240 days of inoculation. From the analysis of the 21 hybrids, nine of them (H68, H106, H109, H113, H142, H156, H154, H161, and H165) were selected for the subsequent steps. The H109, H161, H165, H113, H154, and H146 hybrids were infected, but the H68, H106, and H142 hybrids were negative for the presence of CLas in all biological replicates (Tables 1 and 2).

**Table 1:** Detection and quantification of the bacteria by quantitative PCR (qPCR) in *Citrus sinensis*, *C. sunki*, *Poncirus trifoliata* and nine hybrids from an F_1_ population obtained from the cross between *C. sunki* and *P. trifoliata* Raf. cv Rubidoux. Each individual is represented by five repetitions.

Genotypes	HLB diagnosis (qPCR) days after inoculation				
	30	90	180	240	360
*C. sunki*	0/5	3/5	4/5	4/5	5/5
*C. sinensis*	0/5	3/5	5/5	5/5	5/5
*P. trifoliata*	0/5	0/5	0/5	3/5	3/5
H106	0/5	0/5	0/5	0/5	0/5
H109	1/5	2/5	4/5	5/5	5/5
H146	1/5	1/5	3/5	5/5	5/5
H68	0/5	0/5	0/5	0/5	0/5
H161	0/5	3/5	5/5	5/5	5/5
H142	0/5	0/5	0/5	0/5	0/5
H165	0/5	3/5	5/5	5/5	5/5
H154	–	–	4/5	5/5	5/5
H113	–	–	5/5	5/5	5/5

–: Correspond to samples that were not evaluated due to the absence of leaves.

**Table 2:** *Candidatus* Liberibacter asiaticus (CLas) quantification obtained by comparing the standard curve of the HLB primers with the standard curve of the internal control gene (GAPDH) initiators. The value quantification refers to Log_10_ of the number of copies of the CLas fragment after 240 days from inoculation in each repetition per genotype included in RNAseq analysis.

Genotype	Ct value of GAPDH	Ct value of HLB	Quantification/Log_10_ number of copies
*C. sinensis*	24.24	26.48	3.48
	22.07	24.30	4.09
	20.12	31.74	2.01
*C. sunki*	18.43	24.90	3.92
	18.18	20.51	5.15
	18.56	25.17	3.85
*P. trifoliata*	19.99	20.31	5.20
	19.20	21.85	4.78
	18.30	25.80	3.67
H109	19.16	17.95	5.86
	18.30	20.35	5.19
	19.13	20.38	5.19
H161	20.32	21.12	4.98
	19.28	18.87	5.61
	19.23	18.75	5.64
H165	18.98	18.85	5.61
	19.05	22.18	4.68
	18.52	18.87	5.61
H113	17.67	21.93	4.75
	19.14	26.25	3.55
	19.05	20.81	5.07
H154	19.47	30.79	2.28
	19.05	19.33	5.48
	19.05	20.55	5.14
H146	18.78	21.33	4.92
	18.35	22.16	4.69
	18.48	25.60	3.73
H68	20.28	Undetermined	0
	19.39	Undetermined	0
	19.50	Undetermined	0
H106	19.96	Undetermined	0
	19.86	Undetermined	0
	18.34	Undetermined	0
H142	20.93	Undetermined	0
	18.59	Undetermined	0
	18.99	Undetermined	0

### Phenotypic analysis

**Figure 1:**
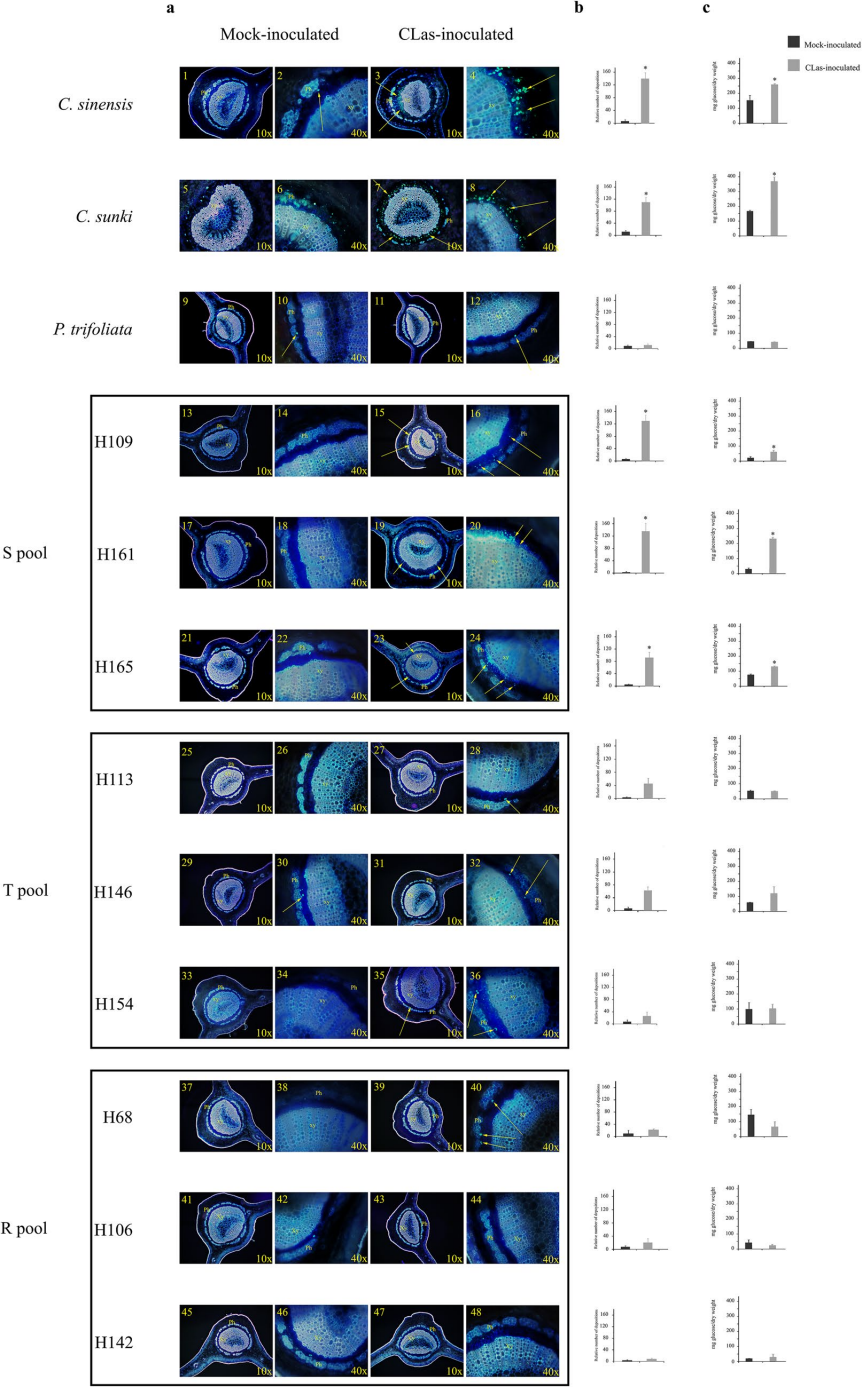
Callose deposition. (**a**) Cross sections of leaf petioles of *C. sinensis* mock-inoculated (1 and 2) and CLas inoculated (3 and 4), *C. sunki* mock-inoculated (5 and 6) and CLas inoculated (7 and 8), *P. trifoliata* mock-inoculated (9 and 10) and CLas inoculated (11 and 12), H109 mock inoculated (13 and 14) and CLas inoculated (15 and 16), H161 mock-inoculated (17 and 18) and CLas inoculated (19 and 20), H165 mock-inoculated (21 and 22) and CLas inoculated (23 and 24), H113 mock-inoculated (25 and 26) and CLas inoculated (27 and 28), H146 mock-inoculated (28 and 30) and CLas inoculated (31 and 32), H154 mock-inoculated (33 and 34) and CLas inoculated (35 and 36), H68 mock-inoculated (37 and 38) and CLas inoculated (39 and 40), H106 mock-inoculated (41 and 42) and CLas inoculated (42 and 44), H142 mock-inoculated (45 and 46) and CLas-inoculated (47 and 48). FL, phloem; Xi, xylem. (**b**) The bar graph next to the microscopy plates show the callose quantification performed by counting fluorescent spots marked by aniline blue dye. Quantification was performed with tree replicates per genotype, inoculated plants (positive or negative HLB) and mock-inoculated plants. (**c**) Starch quantification. Individuals were inoculated with CLas (CLas-infected) or mock-inoculated (CLas-free) and collection was performed after 240 days, and quantification was carried by the enzymatic method. Bars represent the standard deviation between 3 biological replicates. * *p*_value < 0.05 (Mock-inoculated × CLas inoculated).

A significant increase in callose deposition was observed for the CLas-infected *C. sinensis*, *C. sunki,* H109, H161, and H165 plants compared to the control (Fig. 1). Moreover, *P. trifoliata,* H113, H154, H146, H68, H106, and H142 showed no difference between the mock and CLas-inoculated plants (Fig. 1). Compared with inoculated and mock-inoculated plants, *C. sinensis, C. sunki,* and three infected hybrids (H109, H161 and H165) showed a significant difference (*p* < 0.05) in the amount of starch. In contrast, no significant difference in starch accumulation was observed in *P. trifoliata* and the other six hybrids (H113, H154, H146, H68, H106, and H142) (Fig. 1).

In general, the visual symptoms were more evident in the susceptible plants, while the visual HLB symptoms were undefined in *P. trifoliata* and its hybrids. However, according to CLas detection, starch and callose quantification between different treatments, the hybrids were clustered into three distinct groups as follows: Susceptible Pool (S Pool), composed of three different hybrids (H109, H161, and H165) that were diagnosed as HLB-positive and presented elevated starch and callose deposition, similar to that observed for susceptible parental genotypes (Fig. 1); Tolerant Pool (T Pool), composed of three different hybrids (H113, H154, and H146) that were diagnosed as HLB-positive but did not exhibit a significant starch and callose accumulation as observed in susceptible genotypes (Fig. 1); and Resistant Pool (R Pool), composed of three different hybrids (H68, H106, and H142) that were diagnosed as HLB-negative with starch quantification similar to healthy plants (mock-inoculated plants) (Fig. 1).

### Transcriptome assembly

To elucidate the different responses to CLas infection, we studied the changes in global transcriptional level in susceptible, tolerant, and resistant genotypes infected by CLas. In this work, 36 cDNA libraries from six different genotypes of either CLas-inoculated or mock-inoculated (control) samples were evaluated. After trimming, 487 million reads were obtained, and 95% of the total was assigned (see Supplementary Table S1). The reads were mapped in 133,976 transcripts on the *C. sinensis* genome available on http://citrus.hzau.edu.cn/.

HLB-susceptible genotypes, *C. sinensis* and *C. sunki*, showed a high number of differentially expressed genes (6141 and 5624 DGEs, respectively) compared with the tolerant parental, *P. trifoliata* (100 DEGs) (Table 3). A similar pattern was observed between the pool of hybrids. The S Pool showed 708 differentially expressed genes (DEGs), while the R Pool presented only 92 DGEs. The Tolerant Pool (T Pool) showed the highest number of DEGs (2027) among the hybrid pools. Most of these genes were downregulated in HLB-infected plants compared with healthy ones (Table 3).

**Table 3:** Number of differentially expressed genes in *C. sinensis*, *C. sunki*, *P. trifoliata*, S Pool, T Pool and R Pool. CLas-infected plants compared with healthy plants.

Genotypes	Up-regulated	Down-regulated	Total
*C. sinensis*	3175	2966	6141
*C. sunki*	3288	2336	5624
*P. trifoliata*	70	30	100
S Pool	288	420	708
T Pool	939	1088	2027
R Pool	63	29	92
Total	5812	5331	14,692

The principal component analysis (PCA) using the Bioconductor package (see Supplementary Fig. S1) showed the replicates of the different genotypes in general grouped according to the analyzed condition for *C. sunki*, *C. sinensis*, and the susceptible and tolerant hybrids. The resistant groups in fact presented a mixed grouping, which is not surprising if we consider that these hybrids were the ones that showed the fewer number of DEGs. The genotype grouping indicated that the global expression landscape is related more to the different genotypes and not the analyzed condition (infection by CLas). In this case, the identification of genes exclusively differentially expressed in the genotypes considered susceptible, tolerant, or resistant as well as genes that had antagonistic expression between the opposite phenotypes became important to increase our understanding of the different responses.

### Differential gene expression analysis

**Figure 2:**
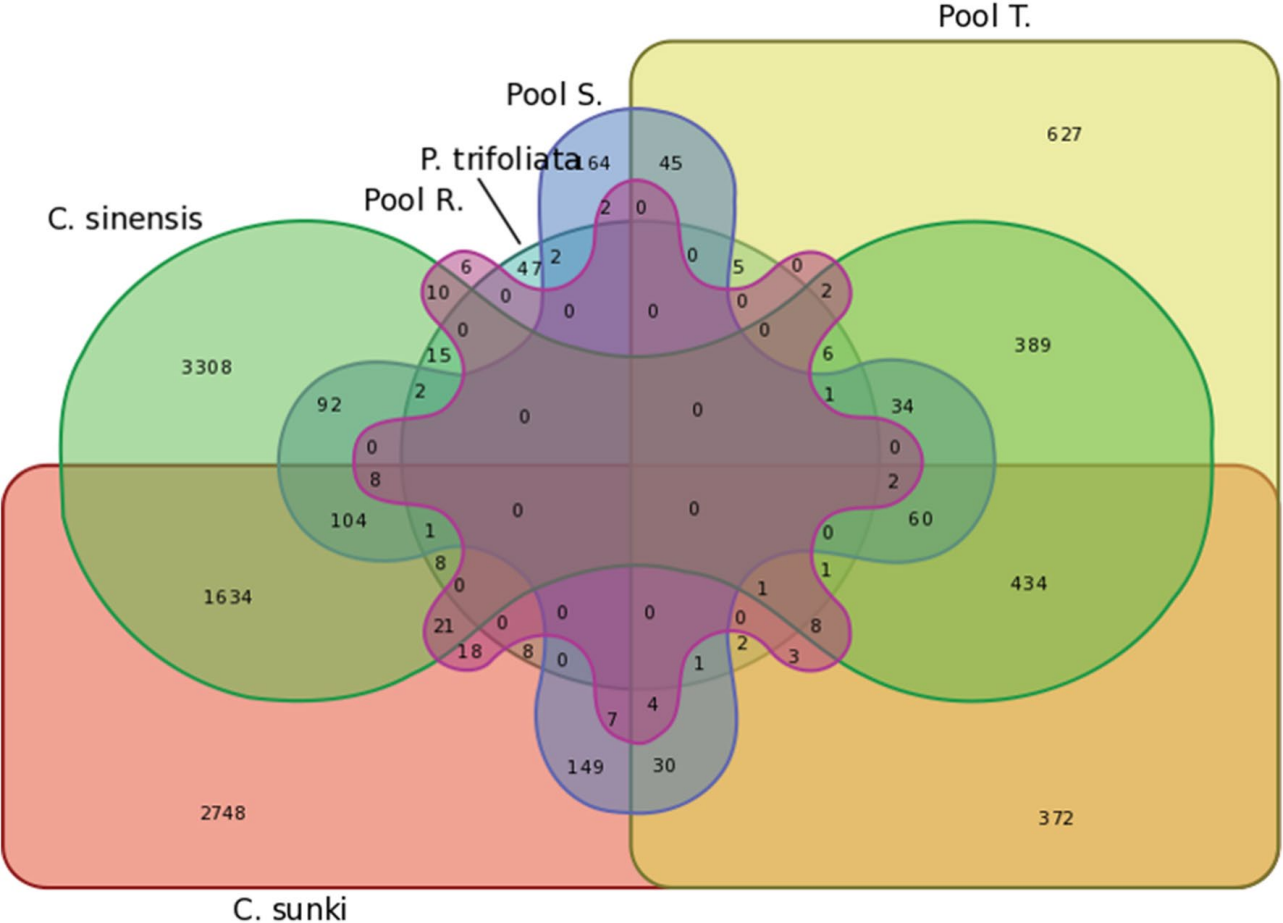
Venn diagram, considering common and exclusive DGEs of *C. sinensis*, *P. trifoliata, C. sunki*, S Pool, T Pool and R Pool.

The results are summarized in a Venn diagram (Fig. 2 and Table S2). The susceptible genotypes, *C. sinensis* and *C. sunki*, exhibited the highest number of overlapping DEGs (1634), and 88% of these genes presented a similar expression pattern (Fig. 2, Supplementary Table S2), suggesting that a similar gene modulation is caused by CLas infection. In *P. trifoliata,* 47% of the DGEs were exclusive of this genotype (Fig. 2 and Supplementary Table S2), and 26% of the DGEs were overlapped and showed antagonistic expression compared to susceptible genotypes. Five of the downregulated genes in *P. trifoliata* were upregulated in both *C. sinensis* and *C. sunki* genotype, and one gene was upregulated in the S Pool (see Supplementary Table S3 and S4).

Among the seven genes upregulated in the R Pool, five were downregulated in *C. sinensis*, and one gene was downregulated in the T Pool and another one in *C. sunki* (see Supplementary Table S5). The study of genes with antagonistic expression between susceptible and tolerant and/or resistant genotypes may help to explain possible tolerance mechanisms as well as to identify good targets for plant resistance.

### Main processes affected by CLas infection

**Figure 3:**
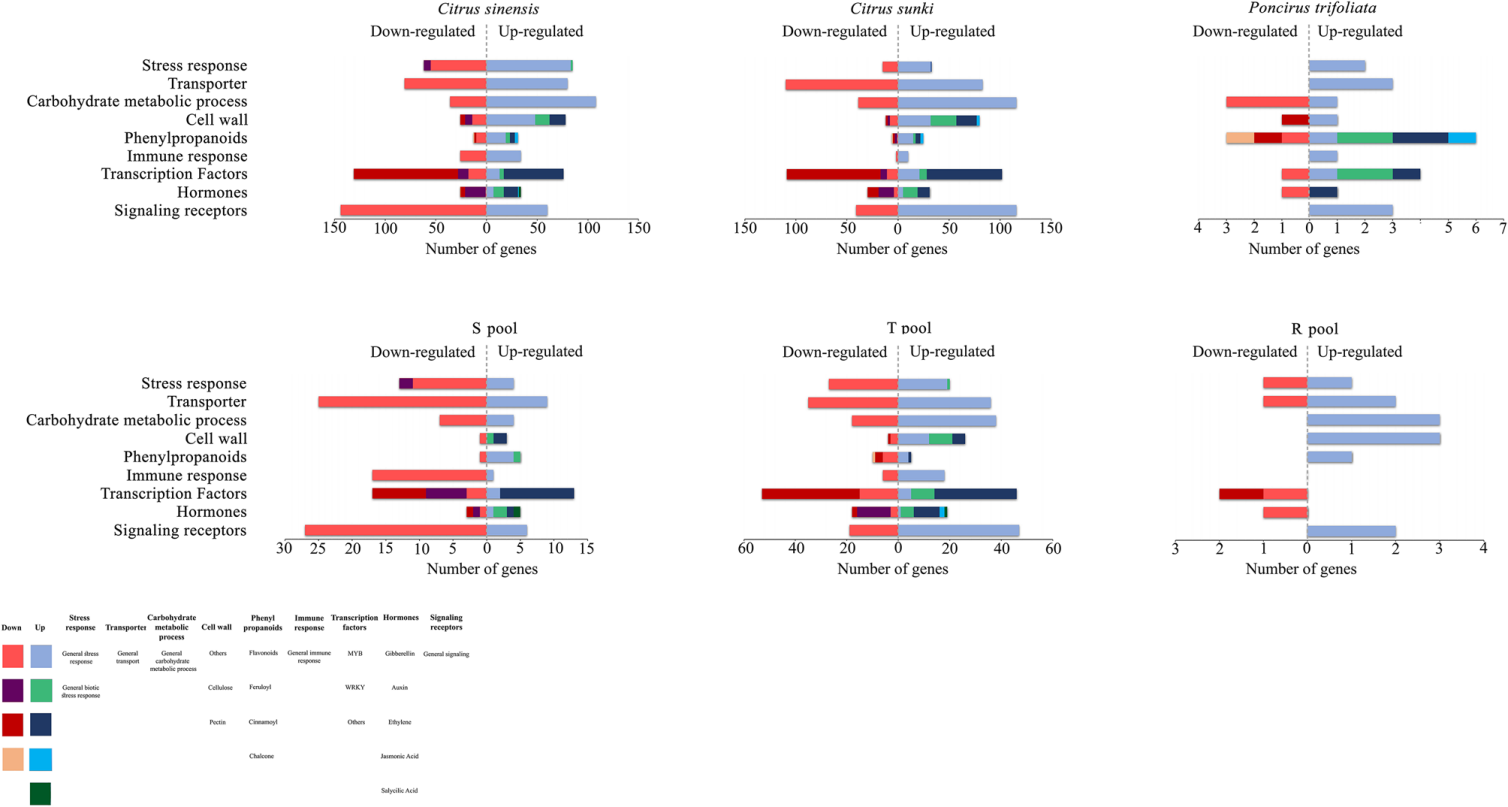
*C. sinensis*, *C. sunki*, *P. trifoliata*, S Pool, T Pool and R Pool responses to 240 days of infection by CLas. Genes are classified into nine groups (Stress response, Transporter, Carbohydrate metabolic process, Cell wall, Phenylpropanoids, Immune response, Transcription Factors Hormones and Signaling receptors) according to Blast2GO analysis and based on their expression pattern. The number of down-regulated genes in response to CLas is represented by the bars in reddish tones and upregulated in blue tones. Some bars present subdivisions and the color legend for each pathway is indicating the specific, related gene or specific pathways, which were important to illustrate the proposed tolerance mechanism to HLB.

Libraries of DEG functions assigned by Blast2GO^9^ and Gene ontology (GO)^10^ analyses helped us better understand the differences in genetic responses involved in susceptibility, tolerance, or resistance (Fig. 3). Susceptible genotypes and tolerant hybrids differentially expressed many genes in comparison to resistant hybrids and *P. trifoliata*. These different pathways provided valuable information regarding the genetic mechanisms of CLas perception and responses activated in tolerant/resistant and susceptible hosts (Fig. 3).

### Differentially expressed genes (DEGs) associated with a specific biological pathway

#### Signaling receptor

Plant receptors are responsible for the recognition of several external stimuli, including pathogen attack. These transmembrane proteins are directly associated with signaling pathways, which trigger a proper physiological response^11^. Several types of receptors were regulated in *C. sinensis*, *C. sunki*, the S Pool, and the T Pool, and most of them were downregulated in those genotypes (Fig. 3). In *P. trifoliata* and the R Pool only a few receptors were differentially expressed, and most of them were induced (Fig. 3). These receptors included *G-type lectin S-receptor-like, cysteine-rich receptor kinase,* and *serine/threonine-protein kinase,* which were upregulated in *P. trifoliata,* and *leucine-rich repeat transmembrane kinase* and *leucine-rich repeat receptor-like protein kinase,* which were induced in the R Pool (see Supplementary Table S6). Therefore, our results suggests that downregulation of receptors may be associated with susceptible response to CLas.

#### Hormones

Genes associated with auxin and ethylene pathways were barely or not affected in *P. trifoliata* and the R Pool, whereas many auxin and ethylene-related genes were differentially expressed in *C. sunki*, *C. sinensis*, the T Pool, and the S Pool under CLas infection. Interestingly, no important changes in the transcriptional profiles of genes related to SA and JA biosynthesis were found (Fig. 3). In addition, CLas induced key genes involved with gibberellin (GA) degradation in tolerant and resistant genotypes, while the related GA synthesis genes were downregulated. In *P. trifoliata*, the *gibberellin-induced* gene was one of the top three downregulated DEGs (log2 fold change =  − 10) (see Supplementary Table S6). The opposite pattern was observed in CLas-susceptible genotypes, in which an induction of genes involved with GA synthesis and downregulation of GA degradation was observed. Thus, these findings suggests that GA plays an important role in CLas-citrus interactions, affecting plant physiology and consequently HLB symptoms.

#### Transcription factors

Plant responses to pathogen attack require large-scale transcriptional reprogramming. *P. trifoliata* showed only five transcription factor (TF)-related genes modulated by CLas infection. Only the MYB TF was downregulated. The other four TFs were upregulated, including two WRKY TFs (Fig. 3). The resistant hybrids suppressed the expression of another class of transcription factor, the *SCL domain* (see Supplementary Table S6). In contrast, hundreds of TF genes showed changes at the transcription level in *C. sinensis*, *C. sunki*, the S Pool and the T Pool (Fig. 3). In this context, the large number of TFs affected in these genotypes may be directly related to the regulation of genes responsive to HLB infection. Of note, several WRKY TFs were identified in *C. sinensis* and *C. sunki,* and most of them were repressed in CLas-infected plants (Fig. 3). Therefore, these results indicated that the increase in transcription of WRKY TFs in *P. trifoliata* is associated with the genetic defense mechanism involved with HLB tolerance.

#### Defense-related genes

Defense-related genes are directly related to processes or production of compounds able to inhibit pathogen reproduction or to make further infection more difficult^12^. In particular, one defense-related gene, e*ndochitinase B*, was differentially expressed and highly upregulated in resistant hybrids (see Supplementary Table S6). Endochitinases have previously been reported as important bactericides, and some of them have ability to cleave peptidoglycan chains, promoting bacterial cell lysis^13^. Other defense-related genes were differentially expressed in susceptible plants by CLas. Among them, regions encoding lipid transfer, molecular factors that help the innate immune system of plants, and small lipid-transfer proteins can inhibit fungal growth and pathogenic bacteria^14^. Genes encoding these proteins were differentially expressed in *C. sinensis*, *C. sunki,* and the S Pool (see Supplementary Table S6 and Fig. S2). These results indicated the activation of defense pathways in response to CLas infection in susceptible genotypes.

CDR1 also represents an important defense related gene in *Poncirus* and *Poncirus*-hybrids^15^. CDR1 showed high expression in all the Poncirus hybrids, including the S pool, but it was only induced in the R pool. Therefore, even though it could be associated with resistance, high CDR1 constitutive expression level seems not to be sufficient to lead to the resistance phenotype.

#### Secondary metabolism and cell wall composition

Secondary metabolites often play an important role in many physiological responses, such as growth, photosynthesis, reproduction, and plant defenses against pathogens^16^. The most upregulated genes in *P. trifoliata* included a variety of phenylpropanoids and lignin-related genes, such as *caffeic acid O-methyltransferase, chalcone synthase, feruloyl ortho-hydroxylase 1, hydroxycinnamoyl transferase* and *laccase precursor* (see Supplementary Fig. S2). In our study, the *laccase precursor* gene, whose protein catalyzes lignin and its derivatives^17^, was exclusive and highly induced in CLas-infected *P. trifoliata* (see Supplementary Table S6).

Pectin hydrolysis occurs frequently in response to bacterial infection^18^. Just one pectin degradation-related gene was differentially expressed (downregulated) in *P. trifoliata* (Fig. 3 and Supplementary Table S6). Many genes involved in pectin synthesis and degradation were differentially expressed in *C. sinensis* and *C. sunki.* Pectin methyltransferases are enzymes that induce pectin modification. In *C. sinensis* and *C. sunki* under stress caused by CLas infection, the *pectin methyltransferase 1* gene was upregulated (see Supplementary Fig. S2).

A larger number of DEGs involved in cellulose synthesis showed mRNA levels altered in susceptible genotypes; however, *P. trifoliata* and the R Pool did not exhibit differentially expressed regions encoding cellulose (see Supplementary Table S6).

These results demonstrated that the cell wall is highly affected in susceptible plants even at 240 days after CLas inoculation. At the same time, genes involved in cell strengthening proved to be important in *P. trifoliata*.

#### Phloem-related genes

It is already known that callose deposition and phloem proteins (PP2) act as a physical barrier, attempting to block systemic spread of CLas; however, they also likely cause phloem disorders^19^. The current study identified DEGs coding phloem proteins that had altered expression induced by CLas in *C. sinensis*, *C. sunki*, the S Pool, and T Pool. Although *P. trifoliata* did not present callose-induced phloem blockage (Fig. 1), we observed modulation of PP2-B15 in response to CLas with ninefold higher expression than the control (see Supplementary Table S6). That result suggests that *P. trifoliata* modulates phloem genes in response to CLas without over-deposition of callose, consequently not causing important phloem function disorders. Anatomical divergences between *P. trifoliata* and *Citrus* may represent an important feature to avoid collapse of the sieve tube elements^20^.

As shown by our phenotypic data, only susceptible plants had affected callose deposition. Different callose synthases were differentially expressed in the susceptible plants, whereas those genes were absent in *P. trifoliata* and the R Pool inoculated with CLas (see Supplementary Table S7).

Interestingly, genes encoding *sieve element occlusion c* (*SEOc)* and *d* (*SEOd*), which are part of a protein family that encodes specialized crystalloid phloem proteins^21^, were largely upregulated in all susceptible plants under study. Some of these genes were also upregulated in tolerant hybrids (see Supplementary Table S6 and S7).

#### Carbohydrate metabolism

Carbohydrate metabolism was the biological function most affected by HLB (Fig. 3). In the presence of CLas, susceptible genotypes overexpressed genes involved with starch synthesis and suppressed genes that encode enzymes for starch degradation (see Supplementary Table S8 and S9). This phenomenon was not observed for the tolerant and resistant genotypes. Several DEGs involved in the metabolism of starch were identified in *C. sinensis*, *C. sunki,* and the T Pool, especially in the former two (see Supplementary Table S6). Genes encoding *ADP-glucose pyrophosphorylase* and *starch branching enzyme II*, which participate in the synthesis of starch and starch granules, were upregulated in *C. sinensis* and *C. sunki* (see Supplementary Table S6 and S8). Beta and alpha-amylase, important enzymes for normal degradation of the starch in plants^22^, also had their genes expression modulated in both susceptible plants (*C. sinensis* and *C. sunki*) and the T Pool (see Supplementary Table S9). Corroborating our phenotypic data (Fig. 1), resistant and tolerant genotypes did not exhibit altered expression of the main genes involved in synthesis of starch (see Supplementary Table S6). While the R Pool had only *beta-amylase-*encoding gene upregulated, *P. trifoliata* did not have any DEGs related to synthesis and degradation of starch (see Supplementary Table S6).

#### Transporters

The transport of substances was also one of the main biological functions affected by CLas. The transcription levels of genes related to transporters were overwhelmingly altered by CLas infection in all genotypes and hybrids (Fig. 3). In general, susceptible plants had the greatest number of transport-related genes affected by CLas (Fig. 3). The R Pool showed few DEGs related to transport function, including *ABC transporter family, phosphate transporter (PHO1-2),* and *amino acid transmembrane transport* (Supplementary Table 2). Zinc transporter (*ZIP1* and *ZIP8*) genes were differentially expressed in *C. sinensis*, *C. sunki,* and the T Pool (see Supplementary Table S6). Most transport family genes affected by CLas infection were involved with transport of sugars, amino acids, and ions (see Supplementary Table S6). When comparing the transporter-related DEGs in the tolerant genotypes, *P. trifoliata,* and the T Pool, we observed different responses among them. The T Pool exhibited 73 differentially expressed transporter-related genes. The parental *P. trifoliata* showed only 4 differentially expressed transporter-related genes, among which *potassium transporter* was exclusively differentially expressed in *P. trifoliata* (Fig. 3 and Supplementary Table S6).

## Discussion

The hybrids evaluated in this work and the parents, *Citrus sunki* and *P. trifoliata*, were classified as susceptible, tolerant, or resistant according to bacterial presence, callose deposition, and starch accumulation (Fig. 1). RNAseq data indicated that the genotypes responded differently under CLas infection, which was confirmed by RT-qPCR analysis. Overall, the genes showed similar patterns in the RNAseq and RT-qPCR data, but some divergent values were found, which was similar to other transcriptome studies when the results of different techniques were compared^23^.

Our findings indicated that few genes were differentially expressed according to RNAseq analysis of the tolerant and resistant plants. In contrast, RNAseq analysis of susceptible plants showed transcription modulation of many genes. Resistant and tolerant plants have a tendency to respond more rapidly and vigorously to a pathogen than susceptible plants^12^. It is possible that the resistant hybrids have an early response to CLas presence. Early molecular interactions are well-known mechanisms in plant-pathogen interactions^24–26^. Nevertheless, to verify that the genetic responses were due to CLas infection and to avoid false positives, the samples for transcriptomic analysis were collected 8 months after CLas infection.

*P. trifoliata* showed upregulation of receptor-related genes, which presented an efficient recognition of CLas and possibly an effective signaling and activation of defense response against CLas. The reprogramming of defense signaling pathways has previously been reported as a critical element of the early response to CLas in tolerant genotypes^27^, such as *P. trifoliata*. Previous studies have also highlighted the induction of phenylpropanoid-related genes as a molecular mechanism of HLB tolerance^5^. Lignin-related genes and several phenylpropanoids were strongly upregulated in *P. trifoliata* transcriptome (Supplementary Table S6). As reorganization of plant growth and development are critical to maximize plant survival under stress^28^, cell wall reinforcement is a tolerance mechanism of *P. trifoliata* against CLas. When comparing *P. trifoliata* and resistant hybrids, we observed a distinct transcriptional response to CLas (Fig. 2). However, all replicates of the resistant hybrids did not present any detection of CLas, even after almost 1 year of the experiment (Tables 1 and 2), and probably for this reason, they exhibited few DEGs in RNAseq. Interestingly, the exclusive DEGs of the R Pool, formed by the CLas-negative hybrids, may be linked with genes and mechanisms capable of eliminating the bacteria from the plant, such as *endochitinase B*. Plant endochitinases cleave peptidoglycan chains, thereby promoting bacterial cell lysis^13^.

CLas infection is erratic and unpredictable, and even susceptible plants can escape from infection. Until almost 1 year, all plant replicates classified as resistant did not present CLas titer (Tables 1 and 2). Therefore, until that moment, we considered that those plants were resistant to CLas infection and that a mechanism was utilized to avoid spreading the disease.

In the transcriptome of tolerant genotypes, downregulation of GA synthesis genes and upregulation of genes involved with GA degradation were observed, and the opposite behavior was observed in the susceptible genotypes (induction of GA synthesis and repression of GA degradation). In addition, we observed upregulation of several auxin-induced genes and repression of auxin responsive factors (Supplementary Table S6). It is known that the GA pathway presents cross-talk with auxin and ethylene hormones, which are plant growth regulators that also have been associated with plant defense and microbial pathogenesis^29,30^. The present study showed that these regulators were strongly differentially expressed in the tolerant plants by CLas. It has been reported that auxin induces GA biosynthesis and suppresses GA degradation through modulation of several transcription factors and transporters^31,32^. In citrus-pathogen interactions, crosstalk between auxin and GA has also been reported. Inhibition of GA synthesis promotes inhibition of auxin-induced transcription, consequently reducing symptoms in the citrus-*Xanthomonas citri* interaction^33^.

The plant tolerance mechanism is better explained by the interaction of GA and the salicylic acid (SA) hormone. The GA pathway is considered a hormone modulator of the SA signaling backbone during plant responses to pathogens^34–36^. In *Arabidopsis thaliana*, Alonso-Ramírez et al. (2009)^36^ showed that GAs and the overexpression of GA-responsive genes increase not only the endogenous levels of SA but also the expression of *ics1* and *npr1* genes involved in SA biosynthesis and action, respectively. However, SA-related genes were almost not modulated in the present study, which might be due to the high SA level in the evaluated stage, resulting in the expression of SA synthesis-related genes no longer being necessary as shown by Oliveira et al., 2019^20^. Moreover, it is known that SA accumulation and downstream signaling events are important components of both pathogen‐associated molecular pattern (PAMP)-triggered immunity (PTI) and effector-triggered immunity (ETI)^37,38^ through increasing the expression of WRKY transcription factors. Many WRKY TFs were induced in the tolerant genotypes and affected in the susceptible plants (Fig. 5). WRKY TFs have been considered key regulators of plant defense against many pathogens, including CLas^27^. The function of some WRKY genes remains unexplored, but in some crop species, specific WRKYs promote tolerance or even resistance to biotic and abiotic stresses^27^. Thus, the induction of WRKY TFs may also be related to the activation of genes involved with the tolerance mechanism. For example, in *P. trifoliata,* the *WRKY transcription factor 14–1* was induced, and its orthologue in *Arabidopsis* (known as WRKY22) is an essential component of MAPK-mediated plant defense responses against pathogens. MAPKs are associated with one of the earliest signaling events after plant sensing of PAMPs and pathogen effectors.

Moreover, the tolerant and susceptible genotypes had changes in the level of transcription of many *callose synthases* and *phloem protein* (*PP2*) genes in response to CLas infection (Supplementary Table S6 and S7). In addition, all susceptible plants showed induction of a class of genes that includes the *SEOc* gene (Supplementary Table S7). This class of genes has been reported to encode P-protein subunits^21^. Overexpression of these genes could increase callose and PP2 protein synthesis in the citrus phloem sieve elements. Callose and PP2 accumulation is a crucial factor of phloem blockage in CLas-infected plants^19,39,40^. Phloem blockage causes disturbance of photoassimilate flows from source organs (leaves) to sink organs (roots), resulting in starch accumulation in the leaves as observed in this work and in previous studies^41^.

**Figure 4:**
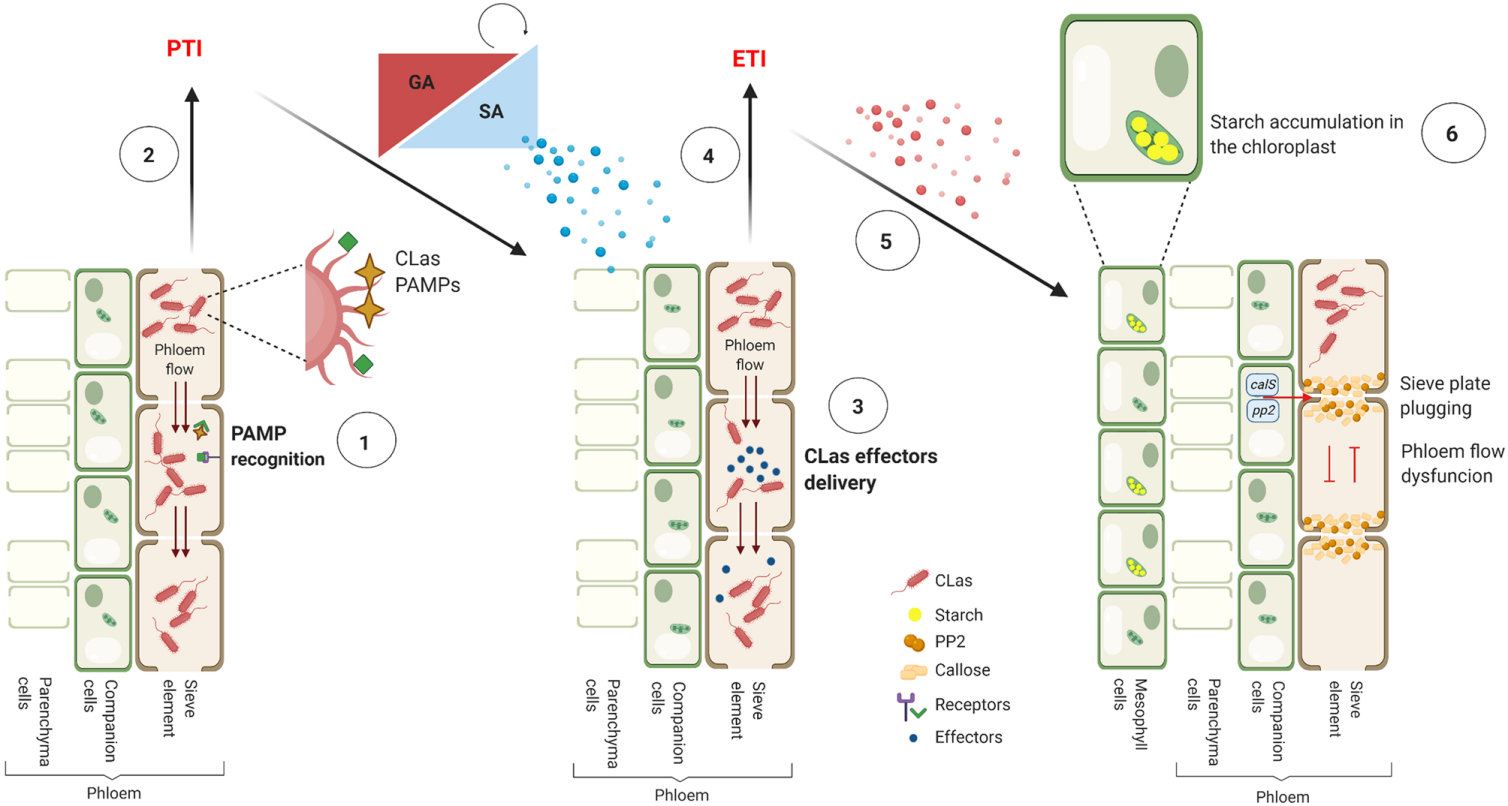
Defense response of susceptible genotypes against CLas. In the phase 1 of this model, citrus plants receptors detect the CLas PAMPs. In phase 2, a PAMP-triggered immunity (PTI) response is initiated, resulting in the production of gibberellic acid (GA), salicylic acid (SA) and the SA-dependent gene expression activation (in blue). In phase 3, CLas deliver effectors leading in effector-triggered susceptibility (ETS). In phase 4, effectors are recognized by plants proteins, activating effector-triggered immunity (ETI). In phase 5, ETI triggers a series of genetic events (in red), including the induction of *calloses synthases* and *pp2* expression. This exaggerated response could be considered as hypersensitive cell death (HR), since the attempt to isolate spatially the CLas leading to callose and PP2 accumulation, that cause phloem dysfunctions. The phase 6 represents the starch accumulation in the mesophyll chloroplasts(Created with BioRender.com).

Based on the knowledge of CLas-susceptible plant interaction that culminates in HLB symptoms, a zig-zag model as illustrated previously by Jones & Dang (2006)^42^ was adapted to explain such genetic molecular response to CLas (Fig. 4). During the beginning of infection, receptors from citrus plants detect the CLas PAMPs, which triggers a PTI response, resulting in the production of GA and SA as well as in the induction of several downstream genes (asymptomatic stage). In a second phase, CLas delivers effectors, such as Las5315^43^ and others^44^, which interfere with PTI or enable pathogen nutrition and dispersal, resulting in effector-triggered susceptibility (ETS). In phase 3, effectors activate an ETI and an amplified version of PTI leading to induction of *callose synthases* and *pp2* gene expression that results in callose and PP2 accumulation. Therefore, callose and PP2 accumulation and the consequent anatomical alterations of the sieve pores may lead to hypersensitive cell death (HR) of the infected plants, which spatially isolate the CLas to reduce their colonizing ability via the phloem^19,40^.

**Figure 5:**
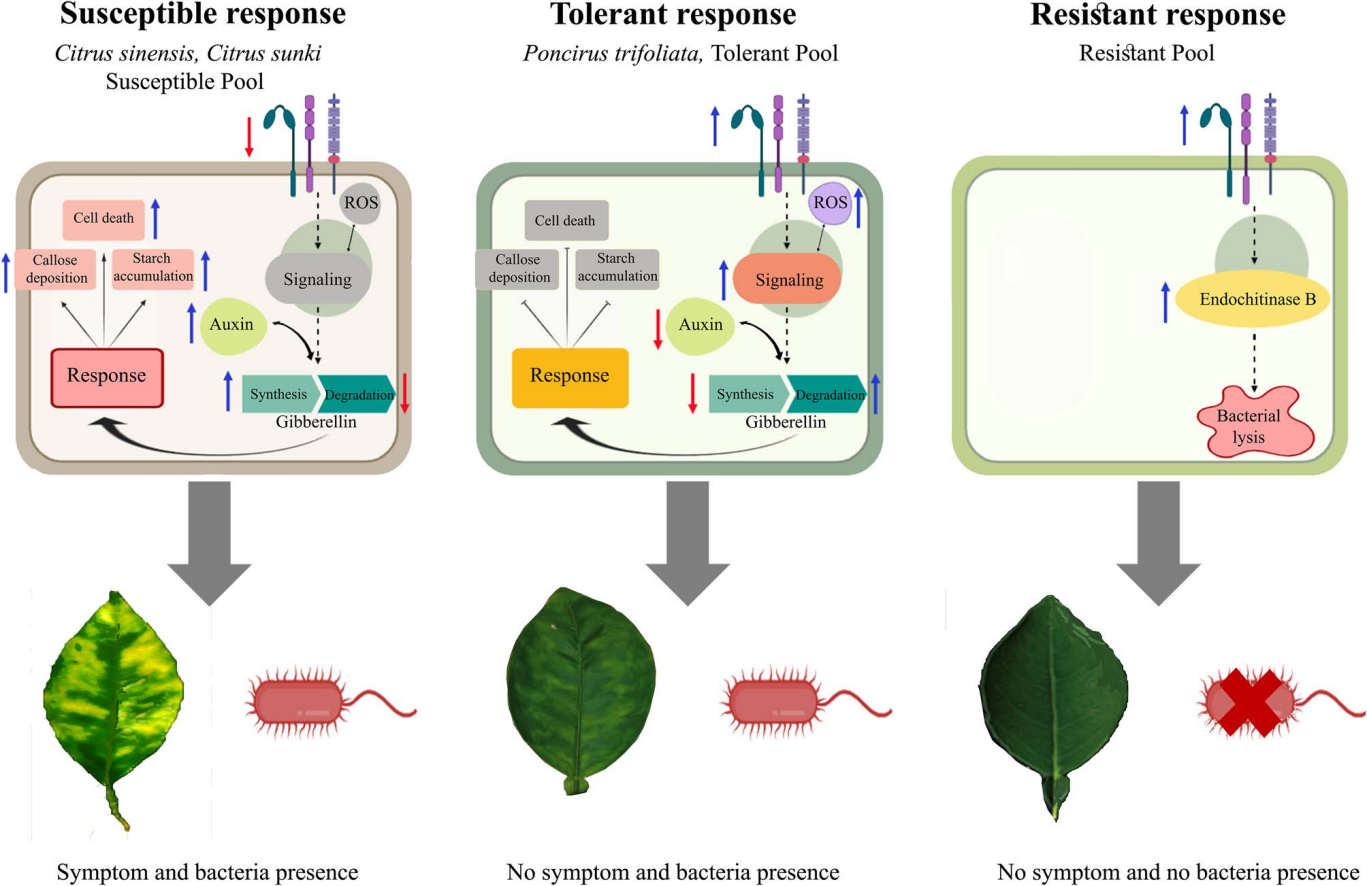
Model of interaction between CLas and Citrus plants. Susceptible plants, the downregulation of signaling receptors promotes a late recognition of CLas infection and consequently, no proper signaling is activated. Auxin-related genes positively modulate the gibberellin synthesis, which activates response mechanisms to CLas infection, such as callose and PP2 deposition and impaired substances transport. Interference on substance transport along with callose deposition causes phloem dysfunction resulting in starch accumulation on photosynthetic tissues. Starch accumulation promotes thylakoid rupture and chlorophyll degradation culminating in HLB classical symptoms. Tolerant plants, the induction of signaling receptors causes a fast and efficient defense response modulated by suppression of auxin pathway and induction of GA degradation. The suppression of these pathways prevents the events that lead to the phloem dysfunction (callose deposition, starch accumulation and transport alteration) and activates defense response through the synthesis of phenylpropanoids and cell wall-strengthened related genes. This transcriptional reprograming is efficient to impair the development of symptoms. Resistant genotypes, a possibly early and fast defense may occur in response to CLas, since low numbers of the genes are modulated after 240 days post inoculation. Nonetheless, this response is related to induction of signaling receptors and upregulation of *Endochitinase B*, which might be associated with bacterial cell lysis (Created with BioRender.com).

To describe the genetic mechanisms potentially involved in a susceptible, tolerant, and resistant interaction with CLas based on the data obtained in this study, we built a hypothetical model (Fig. 5). The model shows that in the susceptible plants (Fig. 5), auxin-related genes positively modulate GA synthesis, which activates response mechanisms to CLas infection, such as callose deposition, PP2 deposition, phloem dysfunction, and impaired flow transport. The impaired flow results in starch accumulation on mesophyll chloroplasts, which promotes thylakoid rupture and chlorophyll degradation, culminating in HLB typical symptoms. In the tolerant plants, including *P. trifoliata* (Fig. 5), the induction of signaling receptors cause a fast and efficient defense response modulated by suppression of the auxin pathway and induction of GA degradation. The suppression of these pathways prevents the events that lead to phloem dysfunction (callose deposition, starch accumulation, and transport alteration), and it activates the defense response through the synthesis of phenylpropanoids and cell wall strengthened-related genes. This transcriptional reprograming is efficient to impair the development of symptoms. In the resistant genotypes (Fig. 5), a potentially early and rapid defense may occur in response to CLas because only a few genes were differentially expressed after 240 days after inoculation. However, this response is related to induction of signaling receptors and upregulation of *endochitinase B*, which is associated with bacterial cell lysis.

Both hypothetical models showed that there are many pathways acting in citrus defense against CLas infection. The data acquired in this study can help to generate citrus varieties of scions or rootstocks with potential resistance to HLB based on citrus conventional breeding programs or biotechnological approaches, including the development of transgenic or cisgenic lines as well as genome editing and host-induced gene silencing.

## Materials and methods

### Plant material

*C. sinensis, C. sunki*, *P. trifoliata,* and 21 hybrids obtained from a controlled cross between *Citrus sunki* ex Tan (female parent and susceptible to HLB) and *Poncirus trifoliata* Raf. cv Rubidoux (male parent and tolerant to HLB) were used in the analysis. *C. sinensis* was included because it is one of most important citrus scions in the world, and it can also be considered an internal control of the experiment considering that *C. sinensis* is characterized as a species highly susceptible to HLB^40^. The experimental design was completely randomized and consisted of five biological replicates for each inoculated genotype (CLas-infected budwoods) and mock-inoculated genotype (health budwoods). Plants were propagated using buds that were grafted onto rootstocks of Rangpur lime (*C. limonia* Osb.). At the end of 6 months, the plant scions were grafted using two *C*Las-infected buds obtained from *C. sinensis* (L.) Osbeck cv Pera. All plants were kept in a greenhouse at Centro de Citricultura Sylvio Moreira of the Agronomic Institute (IAC), SP with an average temperature of 25 °C for 12 months. The starch content and callose deposition were estimated only in the genotypes selected for the further analysis (*C. sinensis*, *C. sunki*, *P. trifoliata,* and 15 hybrids obtained from crosses between *C. sunki* and *P. trifoliata*). Leaves from inoculated and mock-inoculated plants from all evaluated genotypes were collected with three biological replicates of each genotype after 8 months of CLas infection.

### CLas quantification

CLas presence and HLB symptoms were evaluated according to previously described methodology^40^. Briefly, 30, 90, 180, 240, and 360 days after inoculation, to confirm HLB infection, leaves above the inoculation point were collected and tested by qPCR using 16S ribosomal DNA primer sets and FAM/Iowa Black FQ label probe (IDT Inc., Coralville, IA) probes as described by Li et al. (2006)^45^. Citrus GAPDH (glyceraldehyde 3-phosphate dehydrogenase F: GGAAGGTCAAGATCGGAATCAA; R: CGTCCCTCTGCAAGATGACTCT) was used as the reference gene. Values above 34 Ct were considered negative for CLas infection^7^. After 240 days of CLas inoculations, the bacterial titer was evaluated according to Boava et al. (2015)^7^ by qPCR using a standard curve with serial dilutions of 16S ribosomal DNA (rDNA) cloning into pGEM-T vector (PROMEGA).

### Phenotypic analysis

Starch and callose quantification of CLas-inoculated and mock-inoculated plants was performed after 240 days of infection. Callose quantification was performed following the methodology reported previously^40^. Leaf petioles were fixed in FAA solution (50 mL of formaldehyde, 50 mL of glacial acetic acid, and 900 mL of 70% ethanol) for 72 h and then kept in 70% ethanol. Transversal sections of 10 μm were generated using an automatic slide microtome (Leica SM2010R). The sections were stained with blue aniline, and the stained samples were examined on an Olympus BX61 fluorescence microscope using 355–375 nm excitation filter, 400-nm dichromatic mirror, and 435–490 nm emission filter. Callose quantification was performed by counting fluorescent spots in the total phloem area in 10 fields of view for each sample. The starch measurement was performed using leaves dried in an oven at 60 °C for 48 h and ground. Starch content was estimated by enzymatic analysis using 10 mg of dried leaves according to^46^. Absorbance was measured in 96-well microtiter plates using a Microplate Reader (Model 3550–BIO-RAD) at 490 nm. A standard curve was performed using a glucose solution (SIGMA) at concentrations of 0, 2.5, 5.0, 7.5, and 10 μg/mL.

According to starch, callose, and CLas quantification, the genotypes were classified as susceptible, tolerant, and resistant (see supplementary Fig. S3).

### RNA extraction and sequencing (RNA-seq)

Leaves from three biological replicates of the three genotypes (*C. sinensis, C. sunki,* and *P. trifoliata)* and the three hybrid pools (S Pool: H109, H161, and H165; T Pool: H113, H154, and H146; and R Pool: H68, H106, and H142), either CLas-infected inoculated or mock-inoculated plants, were collected for transcriptomic analysis after 240 days of infection. It is difficult to establish the ideal time for studying the first responses and stages of infection because it is difficult to confirm that the plant tissue is colonized by bacteria. Thus, to verify that the genetic responses were due to CLas infection, we performed RNA-seq analysis at 8 months. Total RNA was isolated with the MasterPure Plant RNA Purification Kit (EPICENTRE Biotechnologies, Madison, WI, USA) according to the manufacturer’s instructions. A total of 10 µg of RNA from each sample was sent for sequencing at the Centro de Genômica Funcional in Centro de Biotecnologia Agricola in ESALQ/USP (http://www.esalq.usp.br/genomicafuncional/). RNA-seq was performed using the Illumina HiSeq 2500 platform. All procedures were performed according to Illumina’s protocols. RNA-seq was performed in triplicate with a total of 36 samples.

### Data analysis

The quality of obtained fragments from the sequencing was verified using CLC Genomics Workbench v.6 program (CLC BIO) software (https://www.qiagenbioinformatics.com/products/clc-genomics-workbench/). The sequences were trimmed using the trimmomatic tool^47^ and mapped on the v 2.0 *C. sinensis* genome (http://citrus.hzau.edu.cn/) using the STAR-2.5.2b program^48^. The *R* subread package was used for counting. DEGs between the control and CLas-infected plants were established using the DESeq in Bioconductor package^49^ using an adjusted *p-value* of 0.005 and FDR threshold of 0.05. Venn diagrams (http://bioinformatics.psb.ugent.be/webtools/Venn/) were used to identify common and unique DEGs among the analyzed genotypes. We used Blast2Go^9^ for functional categorization, and the DEGs were annotated by Gene Ontology (GO) using default parameters^10^.

### Real time PCR (RT-qPCR) validation

To ensure reproducibility of the biological phenomenon observed by transcriptomic analysis, we performed a second experiment with other plants following the same design used for RNA-seq. We sampled one hybrid of each pool to represent the susceptible, tolerant, and resistant pools. We used only one hybrid from each pool because it represents the hybrids that comprise each pool regarding CLas infection behavior. Total RNA was extracted using the protocol described by Chang et al. (1993)^50^. Traces of genomic DNA were eliminated using the DNase RNase-Free Ket (QIAGEN, Valencia, CA, USA) according to the manufacturer’s instructions. cDNAs were synthesized from 1.0 μg of total RNA using Superscript III (200 U/μL) (INVITROGEN) with an oligo (dT) primer (dT12-18, INVITROGEN) according to the manufacturer’s instructions.

Ten genes that showed the opposite expression profile between the genotypes with different responses were selected, including *chalcone synthase, lipid transfer, cytochrome P450*, *gibberellin-regulated 9, sieve element occlusion c, cinnamoyl-reductase, pectin methylesterase 1, starch branching enzyme II, PRR response regulator, and choline transporter like-protein 2* (see Supplementary Table S10). Primers were designed using Primer3Plus^51^, and the Primer-BLAST tool^52^ was used to check the specificity of the primers. Two endogenous genes, GAPDH and FBOX, were used for normalization of the data. Relative gene expression was calculated with the 2^−ΔΔCt^ method^53^.

## Supplementary information

Supplementary Figure 1.

Supplementary Figure 2.

Supplementary Figure 3.

Supplementary Table 1.

Supplementary Table 2.

Supplementary Table 3.

Supplementary Table 4.

Supplementary Table 5.

Supplementary Table 6.

Supplementary Table 7.

Supplementary Table 8.

Supplementary Table 9.

Supplementary Table 10.
