# Supplementary material for: Wide-ranging transcriptomic analysis of Poncirus trifoliata, Citrus sunki, Citrus sinensis and contrasting hybrids reveals HLB tolerance mechanisms
Source: Sci Rep. 2020 Nov 30;10:20865. doi: 10.1038/s41598-020-77840-2 (PMC7705011; doi:10.1038/s41598-020-77840-2)
Supplement: Supplementary file 3 — Supplementary Figure 3. [file 41598_2020_77840_MOESM3_ESM.pdf]

# Wide-ranging transcriptomic analysis of *Poncirus trifoliata*, *Citrus sunki*, *Citrus sinensis* and contrasting hybrids reveals HLB tolerance mechanisms

## Supplementary Information

**Author affiliation:**

**Maiara Curtolo**
Centro de Citricultura Sylvio Moreira, Instituto Agronômico de Campinas, Cordeirópolis, São Paulo, Brazil. Universidade Estadual de Campinas, Campinas, São Paulo, Brazil.

**Inaiara de Souza Pacheco**
Centro de Citricultura Sylvio Moreira, Instituto Agronômico de Campinas, Cordeirópolis, São Paulo, Brazil. Universidade Estadual de Campinas, Campinas, São Paulo, Brazil.

**Leonardo Pires Boava**
Centro de Citricultura Sylvio Moreira, Instituto Agronômico de Campinas, Cordeirópolis, São Paulo, Brazil.

**Marco Aurélio Takita**
Centro de Citricultura Sylvio Moreira, Instituto Agronômico de Campinas, Cordeirópolis, São Paulo, Brazil.

**Laís Moreira Granato**
Centro de Citricultura Sylvio Moreira, Instituto Agronômico de Campinas, Cordeirópolis, São Paulo, Brazil.

**Diogo Manzano Galdeano**
Centro de Citricultura Sylvio Moreira, Instituto Agronômico de Campinas, Cordeirópolis, São Paulo, Brazil.

**Alessandra Alves de Souza**
Centro de Citricultura Sylvio Moreira, Instituto Agronômico de Campinas, Cordeirópolis, São Paulo, Brazil.

**Mariângela Cristofani-Yaly**
Centro de Citricultura Sylvio Moreira, Instituto Agronômico de Campinas, Cordeirópolis, São Paulo, Brazil.

**Marcos Antonio Machado**
Centro de Citricultura Sylvio Moreira, Instituto Agronômico de Campinas, Cordeirópolis, São Paulo, Brazil.

**Corresponding author Maiara Curtolo**
Centro de Citricultura Sylvio Moreira, Instituto Agronômico de Campinas, Cordeirópolis, São Paulo, Brazil. Universidade Estadual de Campinas, Campinas, São Paulo, Brazil.
e-mail: maiaramc@hotmail.com

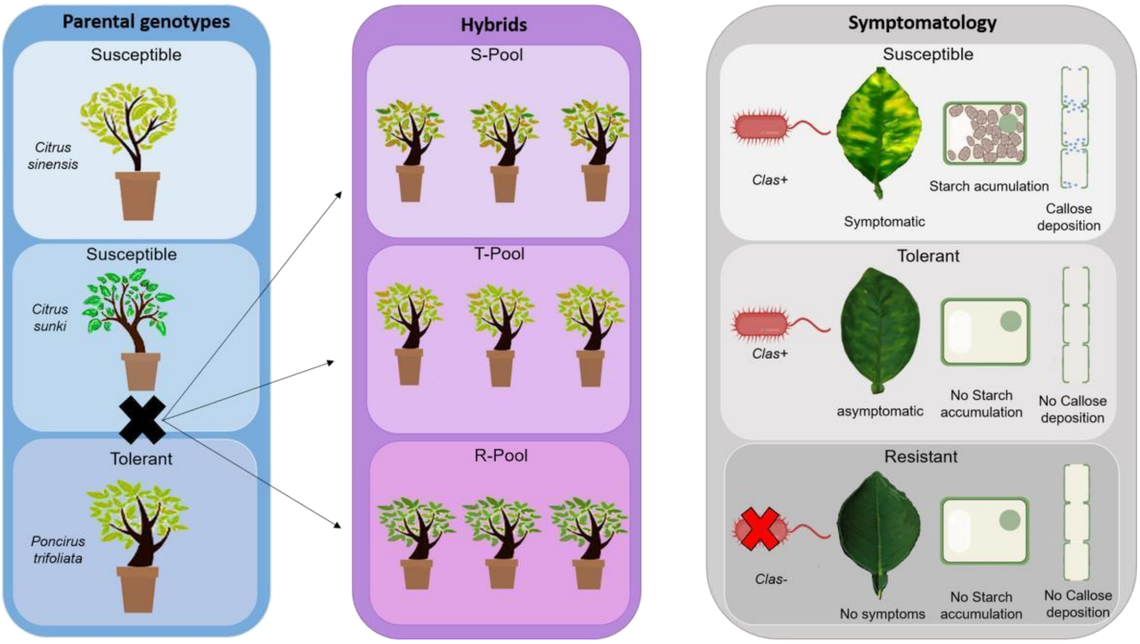


**Supplementary Figure S3:** Pool selection and characterization. Experimental design of hybrid development and symptomatologic standards applied for the determination of different levels of susceptibility, tolerance or resistance observed in parental genotypes and hybrid progeny. The crosses between the susceptible (*C. sunki*) and the tolerant genotype (*P. trifoliata*) generated hybrids with different responses to HLB. Susceptible are those plants that showed both CLas titer and HLB typical symptoms, such as mottle leaves and high accumulation of starch and callose. Tolerant are the plants that showed CLas titer and non-visible HLB symptoms, and no starch and callose accumulation. Resistant are the plants, which presented neither detectable CLas titer nor symptoms or starch and callose accumulation (Created with BioRender.com).
