# Supplementary material for: Wide-ranging transcriptomic analysis of Poncirus trifoliata, Citrus sunki, Citrus sinensis and contrasting hybrids reveals HLB tolerance mechanisms
Source: Sci Rep. 2020 Nov 30;10:20865. doi: 10.1038/s41598-020-77840-2 (PMC7705011; doi:10.1038/s41598-020-77840-2)
Supplement: Supplementary file 4 — Supplementary Table 1. [file 41598_2020_77840_MOESM4_ESM.docx]

**Wide-ranging transcriptomic analysis of *Poncirus trifoliata*, *Citrus sunki, Citrus sinensis* and contrasting** **hybrids reveals HLB tolerance mechanisms**

**Supplementary Information**

**Author affiliation:**

**Maiara Curtolo**

Centro de Citricultura Sylvio Moreira, Instituto Agronômico de Campinas, Cordeirópolis, São Paulo, Brazil. Universidade Estadual de Campinas, Campinas, São Paulo, Brazil.

**Inaiara de Souza Pacheco**

Centro de Citricultura Sylvio Moreira, Instituto Agronômico de Campinas, Cordeirópolis, São Paulo, Brazil. Universidade Estadual de Campinas, Campinas, São Paulo, Brazil.

**Leonardo Pires Boava**

Centro de Citricultura Sylvio Moreira, Instituto Agronômico de Campinas, Cordeirópolis, São Paulo, Brazil.

**Marco Aurélio Takita**

Centro de Citricultura Sylvio Moreira, Instituto Agronômico de Campinas, Cordeirópolis, São Paulo, Brazil.

**Laís Moreira Granato**

Centro de Citricultura Sylvio Moreira, Instituto Agronômico de Campinas, Cordeirópolis, São Paulo, Brazil.

**Diogo Manzano Galdeano**

Centro de Citricultura Sylvio Moreira, Instituto Agronômico de Campinas, Cordeirópolis, São Paulo, Brazil.

**Alessandra Alves de Souza**

Centro de Citricultura Sylvio Moreira, Instituto Agronômico de Campinas, Cordeirópolis, São Paulo, Brazil.

**Mariângela Cristofani-Yaly**

Centro de Citricultura Sylvio Moreira, Instituto Agronômico de Campinas, Cordeirópolis, São Paulo, Brazil.

**Marcos Antonio Machado**

Centro de Citricultura Sylvio Moreira, Instituto Agronômico de Campinas, Cordeirópolis, São Paulo, Brazil.

**Corresponding author**

**Maiara Curtolo**

Centro de Citricultura Sylvio Moreira, Instituto Agronômico de Campinas, Cordeirópolis, São Paulo, Brazil. Universidade Estadual de Campinas, Campinas, São Paulo, Brazil.

Email: maiaramc@hotmail.com

**Supplementary Table S1:** RNA-seq reads and mapping information, C: mock inoculated samples, I: CLas-inoculated samples

| **Genotype** | **HLB** | **Treatment** | **Total reads** | **Unique mapped reads** | **% of Unmapped Reads** |
| --- | --- | --- | --- | --- | --- |
| *C. sinensis* | Susceptible | C | 39,463,048 | 36,194,913 | 2.71 |
|  |  | I | 45,000,006 | 40,458,529 | 3.01 |
| *C. sunki* | Susceptible | C | 39,075,570 | 34,672,395 | 3.87 |
|  |  | I | 45,647,733 | 39,644,055 | 4.23 |
| *P. trifoliata* | Tolerant | C | 46,269,608 | 39,685,082 | 3.86 |
|  |  | I | 41,622,797 | 34,541,507 | 4.49 |
| Pool S | Susceptible | C | 36,266,233 | 31,762,851 | 4.04 |
|  |  | I | 41,328,832 | 36,650,534 | 3.81 |
| Pool T | Tolerant | C | 38,072,190 | 34,012,956 | 3.72 |
|  |  | I | 35,495,818 | 31,859,476 | 3.54 |
| Pool R | Resistant | C | 39,059,147 | 34,931,327 | 3.72 |
|  |  | I | 39,707,545 | 34,582,737 | 4.43 |
