# Supplementary material for: Wide-ranging transcriptomic analysis of Poncirus trifoliata, Citrus sunki, Citrus sinensis and contrasting hybrids reveals HLB tolerance mechanisms
Source: Sci Rep. 2020 Nov 30;10:20865. doi: 10.1038/s41598-020-77840-2 (PMC7705011; doi:10.1038/s41598-020-77840-2)
Supplement: Supplementary file 6 — Supplementary Table 3. [file 41598_2020_77840_MOESM6_ESM.docx]

**Wide-ranging transcriptomic analysis of *Poncirus trifoliata*, *Citrus sunki, Citrus sinensis* and contrasting** **hybrids reveals HLB tolerance mechanisms**

**Supplementary Information**

**Author affiliation:**

**Maiara Curtolo**

Centro de Citricultura Sylvio Moreira, Instituto Agronômico de Campinas, Cordeirópolis, São Paulo, Brazil. Universidade Estadual de Campinas, Campinas, São Paulo, Brazil.

**Inaiara de Souza Pacheco**

Centro de Citricultura Sylvio Moreira, Instituto Agronômico de Campinas, Cordeirópolis, São Paulo, Brazil. Universidade Estadual de Campinas, Campinas, São Paulo, Brazil.

**Leonardo Pires Boava**

Centro de Citricultura Sylvio Moreira, Instituto Agronômico de Campinas, Cordeirópolis, São Paulo, Brazil.

**Marco Aurélio Takita**

Centro de Citricultura Sylvio Moreira, Instituto Agronômico de Campinas, Cordeirópolis, São Paulo, Brazil.

**Laís Moreira Granato**

Centro de Citricultura Sylvio Moreira, Instituto Agronômico de Campinas, Cordeirópolis, São Paulo, Brazil.

**Diogo Manzano Galdeano**

Centro de Citricultura Sylvio Moreira, Instituto Agronômico de Campinas, Cordeirópolis, São Paulo, Brazil.

**Alessandra Alves de Souza**

Centro de Citricultura Sylvio Moreira, Instituto Agronômico de Campinas, Cordeirópolis, São Paulo, Brazil.

**Mariângela Cristofani-Yaly**

Centro de Citricultura Sylvio Moreira, Instituto Agronômico de Campinas, Cordeirópolis, São Paulo, Brazil.

**Marcos Antonio Machado**

Centro de Citricultura Sylvio Moreira, Instituto Agronômico de Campinas, Cordeirópolis, São Paulo, Brazil.

**Corresponding author**

**Maiara Curtolo**

Centro de Citricultura Sylvio Moreira, Instituto Agronômico de Campinas, Cordeirópolis, São Paulo, Brazil. Universidade Estadual de Campinas, Campinas, São Paulo, Brazil.

Email: maiaramc@hotmail.com

**Supplementary Table S3:** Genes down regulated in *P. trifoliata*, which were up regulated in other genotypes

| **Gene description** | **Gene ID** | **Genotype** |
| --- | --- | --- |
| Putative uncharacterized protein Sb01g047790 | orange1.1t00904 | *C. sinensis C. sunki* |
| *flavonoid 3 -monooxygenase-like* | Cs3g05810 |  |
| *nucleic acid-binding* | Cs4g09300 |  |
| *lachrymatory-factor synthase-like* | orange1.1t03813 |  |
| *Chalcone synthase* | Cs2g14720 |  |
| *dihydrofolate reductase* | Cs6g16160 | *C. sinensis* |
| *MYB transcription factor MYB128* | Cs4g13690 |  |
| *unnamed protein product* | Cs4g02690 |  |
| *Licodione synthase* | Cs5g18660 |  |
| *bifunctional 3-dehydroquinate dehydratase shikimate chloroplastic-like isoform X1* | Cs5g32370 |  |
| *kinase 2B* | Cs3g13410 | *C. sunki* |
| *Serine carboxypeptidase-like 18* | Cs8g03880 |  |
| *glutathione S-transferase U8* | Cs6g07260 |  |
| *basic leucine zipper 61* | Cs2g15930 |  |
| *MLP-like protein 423* | Cs7g08260 | S Pool |
