# Supplementary material for: Wide-ranging transcriptomic analysis of Poncirus trifoliata, Citrus sunki, Citrus sinensis and contrasting hybrids reveals HLB tolerance mechanisms
Source: Sci Rep. 2020 Nov 30;10:20865. doi: 10.1038/s41598-020-77840-2 (PMC7705011; doi:10.1038/s41598-020-77840-2)
Supplement: Supplementary file 8 — Supplementary Table 5. [file 41598_2020_77840_MOESM8_ESM.docx]

**Wide-ranging transcriptomic analysis of *Poncirus trifoliata*, *Citrus sunki, Citrus sinensis* and contrasting** **hybrids reveals HLB tolerance mechanisms**

**Supplementary Information**

**Author affiliation:**

**Maiara Curtolo**

Centro de Citricultura Sylvio Moreira, Instituto Agronômico de Campinas, Cordeirópolis, São Paulo, Brazil. Universidade Estadual de Campinas, Campinas, São Paulo, Brazil.

**Inaiara de Souza Pacheco**

Centro de Citricultura Sylvio Moreira, Instituto Agronômico de Campinas, Cordeirópolis, São Paulo, Brazil. Universidade Estadual de Campinas, Campinas, São Paulo, Brazil.

**Leonardo Pires Boava**

Centro de Citricultura Sylvio Moreira, Instituto Agronômico de Campinas, Cordeirópolis, São Paulo, Brazil.

**Marco Aurélio Takita**

Centro de Citricultura Sylvio Moreira, Instituto Agronômico de Campinas, Cordeirópolis, São Paulo, Brazil.

**Laís Moreira Granato**

Centro de Citricultura Sylvio Moreira, Instituto Agronômico de Campinas, Cordeirópolis, São Paulo, Brazil.

**Diogo Manzano Galdeano**

Centro de Citricultura Sylvio Moreira, Instituto Agronômico de Campinas, Cordeirópolis, São Paulo, Brazil.

**Alessandra Alves de Souza**

Centro de Citricultura Sylvio Moreira, Instituto Agronômico de Campinas, Cordeirópolis, São Paulo, Brazil.

**Mariângela Cristofani-Yaly**

Centro de Citricultura Sylvio Moreira, Instituto Agronômico de Campinas, Cordeirópolis, São Paulo, Brazil.

**Marcos Antonio Machado**

Centro de Citricultura Sylvio Moreira, Instituto Agronômico de Campinas, Cordeirópolis, São Paulo, Brazil.

**Corresponding author**

**Maiara Curtolo**

Centro de Citricultura Sylvio Moreira, Instituto Agronômico de Campinas, Cordeirópolis, São Paulo, Brazil. Universidade Estadual de Campinas, Campinas, São Paulo, Brazil.

Email: maiaramc@hotmail.com

**Supplementary Table S5:** Genes up regulated in Pool R which were down regulated in other genotypes

| **Gene description** | **Gene ID** | **Genotype** |
| --- | --- | --- |
| *Quinone oxidoreductase* | orange1.1t00259 | *C. sinensis* |
| *cycloartenol synthase* | Cs4g04730 |  |
| *hypothetical protein VITISV_011279* | Cs1g15880 |  |
| *hypothetical protein VITISV_000078* | orange1.1t03893 |  |
| *sterol regulatory element-binding site 2 protease* | Cs8g03710 |  |
| *uncharacterized protein LOC100795901 precursor* | Cs1g07510 |  |
| *cycloartenol synthase* | Cs4g04680 |  |
| *rubber peroxidase 1* | orange1.1t02045 | T Pool |
| *class IV chitinase* | orange1.1t03118 | *C. sunki* |
