# Supplementary material for: Wide-ranging transcriptomic analysis of Poncirus trifoliata, Citrus sunki, Citrus sinensis and contrasting hybrids reveals HLB tolerance mechanisms
Source: Sci Rep. 2020 Nov 30;10:20865. doi: 10.1038/s41598-020-77840-2 (PMC7705011; doi:10.1038/s41598-020-77840-2)
Supplement: Supplementary file 10 — Supplementary Table 7. [file 41598_2020_77840_MOESM10_ESM.docx]

**Wide-ranging transcriptomic analysis of *Poncirus trifoliata*, *Citrus sunki, Citrus sinensis* and contrasting** **hybrids reveals HLB tolerance mechanisms**

**Supplementary Information**

**Author affiliation:**

**Maiara Curtolo**

Centro de Citricultura Sylvio Moreira, Instituto Agronômico de Campinas, Cordeirópolis, São Paulo, Brazil. Universidade Estadual de Campinas, Campinas, São Paulo, Brazil.

**Inaiara de Souza Pacheco**

Centro de Citricultura Sylvio Moreira, Instituto Agronômico de Campinas, Cordeirópolis, São Paulo, Brazil. Universidade Estadual de Campinas, Campinas, São Paulo, Brazil.

**Leonardo Pires Boava**

Centro de Citricultura Sylvio Moreira, Instituto Agronômico de Campinas, Cordeirópolis, São Paulo, Brazil.

**Marco Aurélio Takita**

Centro de Citricultura Sylvio Moreira, Instituto Agronômico de Campinas, Cordeirópolis, São Paulo, Brazil.

**Laís Moreira Granato**

Centro de Citricultura Sylvio Moreira, Instituto Agronômico de Campinas, Cordeirópolis, São Paulo, Brazil.

**Diogo Manzano Galdeano**

Centro de Citricultura Sylvio Moreira, Instituto Agronômico de Campinas, Cordeirópolis, São Paulo, Brazil.

**Alessandra Alves de Souza**

Centro de Citricultura Sylvio Moreira, Instituto Agronômico de Campinas, Cordeirópolis, São Paulo, Brazil.

**Mariângela Cristofani-Yaly**

Centro de Citricultura Sylvio Moreira, Instituto Agronômico de Campinas, Cordeirópolis, São Paulo, Brazil.

**Marcos Antonio Machado**

Centro de Citricultura Sylvio Moreira, Instituto Agronômico de Campinas, Cordeirópolis, São Paulo, Brazil.

**Corresponding author**

**Maiara Curtolo**

Centro de Citricultura Sylvio Moreira, Instituto Agronômico de Campinas, Cordeirópolis, São Paulo, Brazil. Universidade Estadual de Campinas, Campinas, São Paulo, Brazil.

Email: maiaramc@hotmail.com

**Supplementary Table. S7.** Differentially expressed phloem related in the *C. sinensis*, *C. sunki*, S Pool, T Pool and *P. trifoliata*. ID gene: access number on *C. sinensis* genome.

| **Genotype** | **DGEs** | **ID gene** | **log2FoldChange** |
| --- | --- | --- | --- |
| ***C. sinensis*** | *Callose synthase 5* | Cs1g05830 | -1.57 |
|  | *Plasmodesmata Callose-Binding Protein 3* | Cs5g11770 | 0.89 |
|  | *PP2-B1* | orange1.1t04174 | -1.79 |
|  | *PP2-B10* | Cs2g10930 | 0.94 |
|  | *PP2-A13* | orange1.1t00304 | 1.47 |
|  | *PP2-B1* | Cs9g10910 | 1.73 |
|  | *PP2-A12* | Cs5g10330 | 2.31 |
|  | *PP2-B15* | Cs3g14740 | 3.38 |
|  | *PP2-B15* | Cs3g14720 | 3.40 |
|  | *PP2-B15* | Cs3g14680 | 7.49 |
|  | *PP2-like A1* | Cs7g16020 | 1.11 |
|  | *PP2-like A2* | Cs2g10920 | 3.33 |
|  | *PP2-like B13* | Cs3g14690 | 4.79 |
|  | *Sieve element occlusion c* | Cs5g11280 | 1.18 |
|  | *Sieve element occlusion c* | Cs5g06490 | 2.68 |
|  | *Sieve element occlusion d* | Cs7g09710 | 3.69 |
|  | *Sieve element occlusion d* | Cs2g26900 | 4.23 |
| ***C. sunki*** | *Callose synthase 2* | Cs7g01200 | -1.59 |
|  | *Callose synthase 3* | orange1.1t02029 | 1.24 |
|  | *PP2-B1* | orange1.1t04174 | -2.60 |
|  | *PP2-A12* | Cs5g10330 | 0.82 |
|  | *PP2-B10* | Cs2g10930 | 0.91 |
|  | *PP2-A13* | Cs9g16920 | 1.37 |
|  | *PP2-A13* | orange1.1t00304 | 1.56 |
|  | *Sieve element occlusion c* | Cs5g11280 | 1.48 |
|  | *Sieve element occlusion c* | Cs5g06500 | 1.96 |
|  | *Sieve element occlusion c* | Cs5g06490 | 2.27 |
|  | *Sieve element occlusion d* | Cs7g09710 | 2.94 |
|  | *Sieve element occlusion d* | Cs2g26900 | 3.43 |
| **S Pool** | *PP2-B1* | orange1.1t03219 | -2.06 |
|  | *Sieve element occlusion c* | Cs5g11280 | 1.75 |
| **T Pool** | *Callose synthase 12* | orange1.1t00806 | 1.77 |
|  | *PP2-A13* | Cs9g16920 | -0.83 |
|  | *PP2-A12* | Cs5g10330 | 1.42 |
|  | *PP2-B15* | Cs3g14680 | 7.40 |
|  | *PP2-like B13* | Cs3g14690 | 5.52 |
|  | *Sieve element occlusion c* | Cs5g06500 | 1.82 |
|  | *Sieve element occlusion d* | Cs7g09710 | 2.13 |
|  | *Sieve element occlusion c* | Cs5g06490 | 2.19 |
| ***P. trifoliata*** | *PP2-B15* | Cs3g14680 | 9.81 |
