# Supplementary material for: Wide-ranging transcriptomic analysis of Poncirus trifoliata, Citrus sunki, Citrus sinensis and contrasting hybrids reveals HLB tolerance mechanisms
Source: Sci Rep. 2020 Nov 30;10:20865. doi: 10.1038/s41598-020-77840-2 (PMC7705011; doi:10.1038/s41598-020-77840-2)
Supplement: Supplementary file 11 — Supplementary Table 8. [file 41598_2020_77840_MOESM11_ESM.docx]

**Wide-ranging transcriptomic analysis of *Poncirus trifoliata*, *Citrus sunki, Citrus sinensis* and contrasting** **hybrids reveals HLB tolerance mechanisms**

**Supplementary Information**

**Author affiliation:**

**Maiara Curtolo**

Centro de Citricultura Sylvio Moreira, Instituto Agronômico de Campinas, Cordeirópolis, São Paulo, Brazil. Universidade Estadual de Campinas, Campinas, São Paulo, Brazil.

**Inaiara de Souza Pacheco**

Centro de Citricultura Sylvio Moreira, Instituto Agronômico de Campinas, Cordeirópolis, São Paulo, Brazil. Universidade Estadual de Campinas, Campinas, São Paulo, Brazil.

**Leonardo Pires Boava**

Centro de Citricultura Sylvio Moreira, Instituto Agronômico de Campinas, Cordeirópolis, São Paulo, Brazil.

**Marco Aurélio Takita**

Centro de Citricultura Sylvio Moreira, Instituto Agronômico de Campinas, Cordeirópolis, São Paulo, Brazil.

**Laís Moreira Granato**

Centro de Citricultura Sylvio Moreira, Instituto Agronômico de Campinas, Cordeirópolis, São Paulo, Brazil.

**Diogo Manzano Galdeano**

Centro de Citricultura Sylvio Moreira, Instituto Agronômico de Campinas, Cordeirópolis, São Paulo, Brazil.

**Alessandra Alves de Souza**

Centro de Citricultura Sylvio Moreira, Instituto Agronômico de Campinas, Cordeirópolis, São Paulo, Brazil.

**Mariângela Cristofani-Yaly**

Centro de Citricultura Sylvio Moreira, Instituto Agronômico de Campinas, Cordeirópolis, São Paulo, Brazil.

**Marcos Antonio Machado**

Centro de Citricultura Sylvio Moreira, Instituto Agronômico de Campinas, Cordeirópolis, São Paulo, Brazil.

**Corresponding author**

**Maiara Curtolo**

Centro de Citricultura Sylvio Moreira, Instituto Agronômico de Campinas, Cordeirópolis, São Paulo, Brazil. Universidade Estadual de Campinas, Campinas, São Paulo, Brazil.

Email: maiaramc@hotmail.com

**Supplementary Table. S8.** Differentially expressed related with starch synthesis in *C. sinensis*, *C. sunki*. ID gene: access number on *C. sinensis* genome.

| **Genotype** | **DGEs** | **ID gene** | **log2Fold**  **Change** |
| --- | --- | --- | --- |
| ***C. sinensis*** | *ADP-glucose pyrophosphorylase small subunit* | Cs2g18800 | 1.02 |
|  | *Starch branching enzyme II* | Cs6g15320 | 1.8 |
| ***C. sunki*** | *ADP-glucose pyrophosphorylase family* | Cs5g04870 | 1.17 |
|  | *Starch branching enzyme II* | Cs6g15320 | 0.5 |
