# Supplementary material for: Wide-ranging transcriptomic analysis of Poncirus trifoliata, Citrus sunki, Citrus sinensis and contrasting hybrids reveals HLB tolerance mechanisms
Source: Sci Rep. 2020 Nov 30;10:20865. doi: 10.1038/s41598-020-77840-2 (PMC7705011; doi:10.1038/s41598-020-77840-2)
Supplement: Supplementary file 12 — Supplementary Table 9. [file 41598_2020_77840_MOESM12_ESM.docx]

**Wide-ranging transcriptomic analysis of *Poncirus trifoliata*, *Citrus sunki, Citrus sinensis* and contrasting** **hybrids reveals HLB tolerance mechanisms**

**Supplementary Information**

**Author affiliation:**

**Maiara Curtolo**

Centro de Citricultura Sylvio Moreira, Instituto Agronômico de Campinas, Cordeirópolis, São Paulo, Brazil. Universidade Estadual de Campinas, Campinas, São Paulo, Brazil.

**Inaiara de Souza Pacheco**

Centro de Citricultura Sylvio Moreira, Instituto Agronômico de Campinas, Cordeirópolis, São Paulo, Brazil. Universidade Estadual de Campinas, Campinas, São Paulo, Brazil.

**Leonardo Pires Boava**

Centro de Citricultura Sylvio Moreira, Instituto Agronômico de Campinas, Cordeirópolis, São Paulo, Brazil.

**Marco Aurélio Takita**

Centro de Citricultura Sylvio Moreira, Instituto Agronômico de Campinas, Cordeirópolis, São Paulo, Brazil.

**Laís Moreira Granato**

Centro de Citricultura Sylvio Moreira, Instituto Agronômico de Campinas, Cordeirópolis, São Paulo, Brazil.

**Diogo Manzano Galdeano**

Centro de Citricultura Sylvio Moreira, Instituto Agronômico de Campinas, Cordeirópolis, São Paulo, Brazil.

**Alessandra Alves de Souza**

Centro de Citricultura Sylvio Moreira, Instituto Agronômico de Campinas, Cordeirópolis, São Paulo, Brazil.

**Mariângela Cristofani-Yaly**

Centro de Citricultura Sylvio Moreira, Instituto Agronômico de Campinas, Cordeirópolis, São Paulo, Brazil.

**Marcos Antonio Machado**

Centro de Citricultura Sylvio Moreira, Instituto Agronômico de Campinas, Cordeirópolis, São Paulo, Brazil.

**Corresponding author**

**Maiara Curtolo**

Centro de Citricultura Sylvio Moreira, Instituto Agronômico de Campinas, Cordeirópolis, São Paulo, Brazil. Universidade Estadual de Campinas, Campinas, São Paulo, Brazil.

Email: maiaramc@hotmail.com

**Supplementary Table. S9.** Differentially expressed related with starch degradation in *C. sinensis*, *C. sunki,* S Pool, T Pool and R Pool. ID gene: access number on *C. sinensis* genome.

| ***Genotype*** | **DGEs** | **ID gene** | **log2Fold**  **Change** |
| --- | --- | --- | --- |
| ***C. sinensis*** | *Beta-amylase Family* | Cs5g07550 | -3.9 |
|  | *Beta-amylase 7-like* | orange1.1t00361 | -0.96 |
|  | *Inactive beta-amylase 9* | Cs9g04980 | -1.29 |
|  | *Alpha-amylase 1 large isoform* | Cs3g26820 | 1.25 |
|  | *Alpha amylase domain* | Cs3g23560 | 2.67 |
|  | *Beta-amylase* | Cs2g22040 | 3.29 |
| ***C. sunki*** | *Beta-amylase chloroplastic-like* | orange1.1t03470 | -2.57 |
|  | *Beta-amylase Family* | Cs5g07550 | -2.37 |
|  | *Inactive beta-amylase 9* | Cs9g04980 | -0.95 |
|  | *Alpha-amylase 1 large isoform* | Cs3g26820 | -0.87 |
|  | *Alpha-amylase chloroplastic-like* | Cs7g04310 | -0.65 |
|  | *Alpha amylase domain* | Cs3g23560 | 2.17 |
|  | *Beta-amylase* | Cs2g22040 | 2.21 |
| **S Pool** | *Beta-amylase activity gene* | Cs5g07550 | -1.27 |
| **T Pool** | *Beta-amylase Family* | Cs5g07550 | -1.77 |
|  | *Beta-amylase chloroplastic-like* | orange1.1t03470 | -0.90 |
|  | *Alpha amylase domain* | Cs3g23560 | 2.89 |
| **R Pool** | *Beta-amylase* | Cs2g22040 | 2.61 |
