# Supplementary material for: Wide-ranging transcriptomic analysis of Poncirus trifoliata, Citrus sunki, Citrus sinensis and contrasting hybrids reveals HLB tolerance mechanisms
Source: Sci Rep. 2020 Nov 30;10:20865. doi: 10.1038/s41598-020-77840-2 (PMC7705011; doi:10.1038/s41598-020-77840-2)
Supplement: Supplementary file 13 — Supplementary Table 10. [file 41598_2020_77840_MOESM13_ESM.docx]

**Wide-ranging transcriptomic analysis of *Poncirus trifoliata*, *Citrus sunki, Citrus sinensis* and contrasting** **hybrids reveals HLB tolerance mechanisms**

**Supplementary Information**

**Author affiliation:**

**Maiara Curtolo**

Centro de Citricultura Sylvio Moreira, Instituto Agronômico de Campinas, Cordeirópolis, São Paulo, Brazil. Universidade Estadual de Campinas, Campinas, São Paulo, Brazil.

**Inaiara de Souza Pacheco**

Centro de Citricultura Sylvio Moreira, Instituto Agronômico de Campinas, Cordeirópolis, São Paulo, Brazil. Universidade Estadual de Campinas, Campinas, São Paulo, Brazil.

**Leonardo Pires Boava**

Centro de Citricultura Sylvio Moreira, Instituto Agronômico de Campinas, Cordeirópolis, São Paulo, Brazil.

**Marco Aurélio Takita**

Centro de Citricultura Sylvio Moreira, Instituto Agronômico de Campinas, Cordeirópolis, São Paulo, Brazil.

**Laís Moreira Granato**

Centro de Citricultura Sylvio Moreira, Instituto Agronômico de Campinas, Cordeirópolis, São Paulo, Brazil.

**Diogo Manzano Galdeano**

Centro de Citricultura Sylvio Moreira, Instituto Agronômico de Campinas, Cordeirópolis, São Paulo, Brazil.

**Alessandra Alves de Souza**

Centro de Citricultura Sylvio Moreira, Instituto Agronômico de Campinas, Cordeirópolis, São Paulo, Brazil.

**Mariângela Cristofani-Yaly**

Centro de Citricultura Sylvio Moreira, Instituto Agronômico de Campinas, Cordeirópolis, São Paulo, Brazil.

**Marcos Antonio Machado**

Centro de Citricultura Sylvio Moreira, Instituto Agronômico de Campinas, Cordeirópolis, São Paulo, Brazil.

**Corresponding author**

**Maiara Curtolo**

Centro de Citricultura Sylvio Moreira, Instituto Agronômico de Campinas, Cordeirópolis, São Paulo, Brazil. Universidade Estadual de Campinas, Campinas, São Paulo, Brazil.

Email: maiaramc@hotmail.com

**Supplementary Table. S10.** Primers designed and used for real-time PCR amplification

| Primers |  |  |
| --- | --- | --- |
| *Chalcone Syntase* | F | TCGCCTCGCTAAAGACTTGG |
|  | R | ACCATCACCGAACAAAGCCT |
| *Lipid Transfer* | F | AACCAAGCAAAAGCCTCCCT |
|  | R | AACGCCCTCCAGTTCTCAAG |
| *Cytochrome P450 71A26-Like* | F | GATGATGGAGGCAGTGCAGA |
|  | R | GCAATGGAACTGGTGGGTGA |
| *Gibberellin Regulated 9* | F | CCTGCAGTTTCGATTCACAA |
|  | R | GTGCCTGCAGAAACAGGATT |
| *Sieve Element Occlusion C* | F | GGCGATCCTAGTGTCAGTGG |
|  | R | TCAGCAGTGAAAGGGAAGGC |
| *Cinnamoyl-Reductase* | F | GTGGATGTTAGGGATGTGGCA |
|  | R | GGGTTTTGCTCTTGGGCTCT |
| *Pectin Methylesterase 1* | F | TCTCTCCCGAAAATCCGTGC |
|  | R | GGAAGTGCTGACAGGGAGTT |
| *Starch Branching Enzyme II* | F | AGGTCACCGTCAGCATCTTG |
|  | R | TTATGCCTGTGTCACTGCGT |
| *PRR Response Regulator* | F | CACGGCAGCAATGGACAAAA |
|  | R | CACTATTTCCTGCTGCCCCA |
| *Choline Transporter-Like Protein 2* | F | TGTGTCAGCCTCTCAAGTGC |
|  | R | ACCAAGGAACCAGCAAACCA |
